# Serum from Patients with Severe Alcoholic Liver Cirrhosis Inhibits Proliferation and Migration of Human Coronary Artery Smooth Muscle Cells

**DOI:** 10.3390/jcm10235471

**Published:** 2021-11-23

**Authors:** Mare Mechelinck, Miriam Peschel, Moriz A. Habigt, Daniela Kroy, Michael Lehrke, Marius J. Helmedag, Rolf Rossaint, Matthias Barton, Marc Hein

**Affiliations:** 1 Department of Anesthesiology, Faculty of Medicine, RWTH Aachen University, 52074 Aachen, Germany; mmechelinck@ukaachen.de (M.M.); mipeschel@ukaachen.de (M.P.); mhabigt@ukaachen.de (M.A.H.); rrossaint@ukaachen.de (R.R.); 2 Department of Internal Medicine III, Gastroenterology, Metabolic Diseases and Intensive Care, Faculty of Medicine, RWTH Aachen University, 52074 Aachen, Germany; daniela.kroy@gastroenterologie-aachen.de; 3 Department of Internal Medicine I, Cardiology, Angiology and Internal Intensive Care Medicine, Faculty of Medicine, RWTH Aachen University, 52074 Aachen, Germany; mlehrke@ukaachen.de; 4 Department of General, Visceral and Transplantation Surgery, Faculty of Medicine, RWTH Aachen University, 52074 Aachen, Germany; mhelmedag@ukaachen.de; 5 Molecular Internal Medicine, University of Zürich, 8057 Zürich, Switzerland; barton@access.uzh.ch; 6 Andreas Grüntzig Foundation, 8001 Zürich, Switzerland

**Keywords:** cell proliferation, coronary artery disease, percutaneous coronary intervention, smooth muscle cells, liver cirrhosis

## Abstract

Liver cirrhosis has been associated with an increased risk of coronary artery disease and clinical complications following percutaneous coronary revascularization. The present study is based on the hypothesis that cirrhosis may influence intimal hyperplasia following PCI. Sera from 10 patients with alcoholic liver cirrhosis and 10 age-matched healthy controls were used to stimulate cultured human coronary artery smooth muscle cells (HCASMC) for 48 h. HCASMC proliferation, migration, gene expression and apoptosis were investigated. Serum concentrations of growth factors and markers of liver function were also determined in patients and healthy controls. Treatment of HCASMC with patient sera reduced cell proliferation and migration (*p* < 0.05 vs. healthy controls), whereas apoptosis was unaffected (*p* = 0.160). Expression of genes associated with a synthetic vascular smooth muscle cell phenotype was decreased in cells stimulated with serum from cirrhotic patients (RBP1, *p* = 0.001; SPP1, *p* = 0.003; KLF4, *p* = 0.004). Platelet-derived growth factor-BB serum concentrations were lower in patients (*p* = 0.001 vs. controls). The results suggest the presence of circulating factors in patients with alcoholic liver cirrhosis affecting coronary smooth muscle cell growth. These findings may have implications for clinical outcomes following percutaneous coronary revascularization in these patients.

## 1. Introduction

Liver cirrhosis is associated with an increased risk of coronary artery disease (CAD) [1,2,3,4], just as dyslipidemia, diabetes, renal insufficiency, hypertension and positive family history [5]. However, the latter are used for risk stratification, whereas chronic liver diseases are not part of the European and American guidelines on evaluation and treatment of chronic stable CAD [6,7,8].

Like CAD, end-stage liver disease is associated with low-grade systemic inflammation, including increased levels of molecules related to endothelial activation, acute phase reactants and proinflammatory cytokines [9]. This phenomenon might contribute to the increased prevalence of CAD in patients with liver disease [2]. 

When treating CAD in patients with severe liver cirrhosis, percutaneous coronary intervention (PCI) is the treatment of choice because open bypass surgery is often considered too risky for these patients [10,11]. However, recent analyses suggest that PCI in patients with liver cirrhosis is associated with an increase in mortality, a higher risk of adverse events and higher readmission rates after PCI compared to patients without cirrhosis [12]. Whether the increased risk following PCI is merely due to the high number of known comorbidities and the vulnerability of cirrhotic patients, or whether there are differences in vascular healing compared to non-cirrhotic patients, is not known. 

Smooth muscle cell migration and proliferation are central to atherogenesis and CAD [13,14], and have been identified as contributing factors in restenosis after PCI [14,15].

It has been suggested that the risk of CAD may depend on the etiology of liver cirrhosis. Nonalcoholic steatohepatitis (NASH), for example, has been associated with accelerated development of CAD [1,16,17], whereas the opposite was found in patients with biliary cirrhosis [18]. By contrast, the effect of alcoholic cirrhosis on vascular remodeling, including progression of CAD, is unclear [18,19,20].

The aim of this study, therefore, was to investigate the effects of serum from patients with alcoholic liver cirrhosis on proliferation, migration, apoptosis and steady-state gene expression levels in human coronary artery smooth muscle cells (HCASMC).

## 2. Materials and Methods

The Human Research Ethics Committee at the Universitätsklinikum Aachen approved the use of patient-related data and serum samples (EK166/12). All patients provided informed consent. 

Needed sample size was calculated with a power analysis: from a previous study on the effect of rapamycin on the proliferation of VSMC, an effect size of 2.68 was used [21]. With a power of 0.95 and an alpha error of 0.05, five samples per group are needed to detect a clinically significant difference between groups. However, since the actual effect might be smaller, 10 samples per group were chosen as a safety measure. Therefore, whole blood samples were taken from 10 randomly selected patients with severe alcoholic liver cirrhosis during a routine outpatient examination and 10 age-matched controls. The controls were completely healthy, not hospitalized and all received transient elastography to rule out undetected liver cirrhosis. Serum was prepared by centrifugation at 2000× *g* for 10 min after a clotting time of 15 min and stored at −80 °C [22].

### 2.1. Clinical Chemistry

Bilirubin, aspartate aminotransferase (AST), alanine aminotransferase (ALT), creatinine, C-reactive protein (CRP), interleukin 6 (IL-6), tumor necrosis factor α (TNFα), total cholesterol, low-density lipoprotein (LDL) cholesterol, triglycerides and albumin were measured at the Central Laboratory of the Uniklinik RWTH Aachen.

### 2.2. Measurement of Circulating Growth Factors and Vasoactive Peptides

Concentrations of platelet-derived growth factor (PDGF-BB), angiotensin II, vascular endothelial growth factor (VEGF) and basic fibroblast growth factor (bFGF) were determined in the individual patient sera by ELISAs, pursuant to the kit instructions (PDGF-BB: ab184860, Abcam, Cambridge, UK; bFGF: HSFB00D, R&D Systems, Minneapolis, Minnesota; angiotensin II; ADI-900-204, Enzo Life Sciences, Lausen, Switzerland; VEGF: KHG0111, Life Technologies, Carlsbad, CA, USA). Tests were performed in duplicate.

### 2.3. Human Coronary Artery Smooth Muscle Cell Culture

Commercially available human coronary artery smooth muscle cells (HCASMC) (CC-2583; Lot. No.: 0000289727; third passage; Lonza, Basel, Switzerland) derived from a 57-year-old, non-alcoholic and non-smoking female subject were directly obtained from the company. The cells were thawed following Lonza’s protocol in Smooth Muscle Cell Growth Medium (SmGMTM- 2 BulletKitTM, CC-3182, Lonza) [23]. At passage 5, the medium was changed from SmGMTM- 2 BulletKitTM to Dulbecco’s modified Eagle’s medium (DMEM) (low glucose) (Thermo Fisher Scientific, Waltham, MA, USA, 11885-084) with 10% fetal bovine serum (FBS) (Merck Millipore, Billerica, MA, USA, S0115) and 1% penicillin/streptomycin (Sigma-Aldrich, St. Louis, MO, USA, P0781) (FBS combination) [24]. All experiments were performed with HCASMC at passage 6.

Immunofluorescent staining was performed randomly several times during the experiments: when 80% confluence was reached, the cells were fixed with 2% paraformaldehyde (Otto Fischar GmbH, Saarbrücken, Germany) and permeabilized with 0.2% Triton-X (Sigma-Aldrich, T9284). Non-specific binding sites were blocked with 1% bovine serum albumin (GE Healthcare, Chicago, IL, USA, SH30574) and 1% normal horse serum blocking solution (Vector Laboratories, Burlingame, CA, USA, S-2000). Primary antibodies targeting smooth muscle actin (SMA, mouse monoclonal, anti-human, DAKO Diagnostics Ireland, Ltd., Dublin, Ireland, M0851, dilution 1:200), myosin smooth muscle heavy chain (MYH11, mouse monoclonal, anti-human, Thermo Fisher Scientific, MA5-11648) [25] and transgelin (SM22 alpha, rabbit polyclonal, anti-human, Abcam, ab14106, dilution 1:200) were used [26]. The secondary antibodies used were fluorescein isothiocyanate AffiniPure Goat Anti-Mouse IgG (Jackson ImmunoResearch, Cambridgeshire, UK, 115-095-003, dilution 1:100) for SMA and MYH11 staining and fluorescein isothiocyanate AffiniPure Goat Anti-Rabbit IgG (Jackson ImmunoResearch, 111-095-045, dilution 1:100) for SM22 staining. For mounting, Vectashield Mounting Medium with DAPI (Vector Laboratories, H-1200) was used.

**Figure 1:**
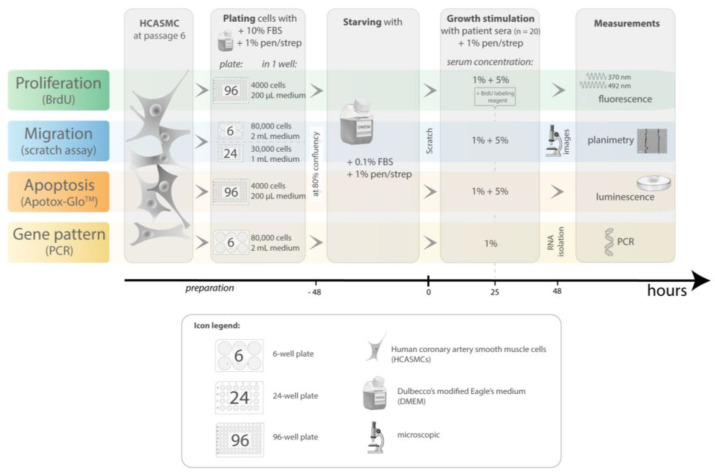
Experimental protocol of the cell culture experiments: proliferation, migration, apoptosis and gene expression in smooth muscle cells after exposure to sera from of patients with alcoholic cirrhosis or healthy controls. All steps including cell plating, starving, growth stimulation and measurements are presented, including details such as seeding-density, type of applied well-plates, serum concentrations used and time course.

Figure 1 provides an overview of the parameters for the experiments on proliferation, migration, apoptosis and steady-state gene expression levels of smooth muscle cells after exposure to patient sera described hereafter (Figure 1).

### 2.4. Basal Cell Growth of Vascular Smooth Muscle Cells

Cultured cells were serum-starved with DMEM, 0.1% FBS and 1% penicillin/streptomycin for 48 h before the experiments [27]. Sera from 10 patients with alcohol-induced cirrhosis of the liver and 10 healthy controls were used at two different concentrations (1% and 5%) in DMEM to stimulate the HCASMC.

### 2.5. Vascular Smooth Muscle Cell Proliferation

For determination of cell proliferation, cells were seeded into 96-well plates with 4000 cells in 200 µL of medium per well. After starvation, the cells were exposed to concentrations of 1% or 5% of the individual sera. All experiments were performed in quadruplicate, and each growth stimulation experiment was carried out twice. A bromodeoxyuridine (BrdU) ELISA (Cell Proliferation ELISA, BrdU (colorimetric), Roche, Basel, Switzerland, 11647229001) was performed in accordance with the kit instructions, and the optional blocking solution was also used (Roche, Basel, Switzerland, 11112589001). Labeling reagent was added 25 h after stimulation, and the optical density of each well was read after 48 h at 370 nm (reference wavelength 492 nm) with a Tecan Microplate Reader (infinite^®^ M200, Tecan Group AG, Männedorf, Switzerland) [28]. 

### 2.6. Apoptosis

Cells were seeded in opaque-walled 96-well culture plates (VWR part of Avantor, Radnor, PA, USA, 732-3225) (20,000 cells per mL and 4000 cells per well), and serum-starved cells were exposed for 48 h to concentrations of 1% and 5% serum. Subsequently, the cells were stained using the ApoTox-GloTM Triplex Assay, according to the manufacturer’s instructions (Promega GmbH, Madison, WI, USA, G6320). Luminescence was measured to quantify apoptosis. The assay was run in duplicate for each individual serum sample [29]. 

### 2.7. Cell Migration

Cell migration was examined using scratch assays [30] in 6- and 24-well plates. Plates were prepared with 2 reference lines at the bottom side of each well to assure that the same sections were repeatedly recorded. At 80% confluence, standard starvation was initiated. Then, a small wound line was made in the cell monolayer—orthogonal to the reference lines—with the tip of a 200 µL pipette. The medium was removed, and the cells were rinsed with HEPES Buffered Saline Solution (Lonza, CC-5024) twice to remove loose cells, followed by incubation with sera at 1% (basal growth) and 5% (stimulated growth). For each well, four microscopic images were taken using transmitted-light microscopy (EVOS XL Cell Imaging System, Thermo Fisher Scientific) 48 h after scratching. The dimensions of the remaining scratch area were calculated planimetrically with ImageJ version 1.49u and the plugin MRI Wound Healing Tool (http://dev.mri.cnrs.fr/projects/imagej-macros/wiki/Wound_Healing_Tool). Migration was calculated by subtracting the basal growth response from the stimulated growth response for each group, and data are given as arbitrary units (AU). Cell counts were set in relation to the healthy controls, as described previously [31,32]. All experiments were performed twice [33].

### 2.8. Steady-State Gene Expression Analysis

HCASMCs seeded in six-well plates underwent a standard adhesion and starvation protocol before each well was stimulated with 1% of one of the 20 individual human serum samples. After 48 h of stimulation, ribonucleic acid (RNA) was isolated from the scraped HCASMCs using a Nucleospin^®^ RNA/Protein Kit following the manufacturer’s instructions (Marcherey-Nagel, Düren, Germany, 740933.250). Complementary deoxyribonucleic acid (cDNA) was produced using a High-Capacity cDNA Reverse Transcription Kit (Life Technologies, 4368814). Quantitative polymerase chain reaction was carried out using TaqManTM Universal PCR Master Mix (Life Technologies, 4304437), an Ribonuclease Inhibitor (Life Technologies, N8080119) and TaqMan probes for the following genes (all from Life Technologies): myocyte enhancer factor 2C (MEF2C) (Hs00231149_m1), Krüppel-like factor 4 (KLF4) (Hs00358836_m1), myocardin (MYOCD) (Hs00538076_m1), secreted phosphoprotein 1 (SPP1 or OPN) (Hs00959010_m1), myosin heavy chain 11 (MYH11) (Hs00975796_m1), retinol binding protein-1 (RBP1) (Hs01011512_g1), serum response factor (SRF) (Hs01065256_m1), transgelin (TAGLN or SM22α) (Hs01038777_g1) and glyceraldehyde phosphate dehydrogenase (GAPDH) (Hs02786624_g1). Real-time polymerase chain reaction analysis was conducted with a StepOnePlus™ Thermocycler (Thermo Fisher Scientific, 15341295). StepOnePlus™ Software v2.3 (Thermo Fisher Scientific) was used for quantification [34]. Individual cycle time values (CT) were normalized to that of the endogenous control GAPDH (∆CT). The relative difference in expression after stimulation with healthy or cirrhotic serum was compared to that of the unstimulated cells (negative control):

∆∆CT = ∆CT stimulated − ∆CT unstimulated,(1)

Relative expression levels of the transcripts are expressed as 2^–∆∆CT^ [35].

### 2.9. Statistics

Data are presented as the mean ± standard error of the mean. Statistical tests were performed using SPSS 24.0 software (IBM Corporation, Armonk, NY, USA), and graphs were generated via GraphPad PRISM 9 software (GraphPad Software, San Diego, CA, USA). Depending on the normality of the distribution and the homogeneity of variances (determined with Levene’s test), one-way analysis of variance (ANOVA) or Kruskal–Wallis tests were used to compare different groups (healthy control group and cirrhotic group). Tukey’s or Dunn’s multiple comparison test was used for post hoc analysis. Group means of cell migration were compared using unpaired *t*-test. Gene expression in relation to that of the negative control (starving) was calculated by a one-sample Wilcoxon test. In addition, two-way ANOVA was used for comparisons of BrdU incorporation, cell migration and apoptosis to test for the effects of group and serum concentration. Comparisons showing significant differences were subsequently analyzed using the Tukey’s post hoc test. A *p* value < 0.05 was considered statistically significant.

## 3. Results

### 3.1. Clinical Parameters of Cirrhotic Patients and Healthy Controls

The main patient characteristics are shown in Table 1. There was no significant difference in mean age (50.9 ± 1.5 years vs. 50.4 ± 2.0 years; *p* = 0.847) or sex (50% vs. 80%; *p* = 0.178) between the two groups (cirrhotic vs. healthy). The 10 patients in the group with alcoholic liver cirrhosis had an average Model for End-Stage Liver Disease (MELD) score of 17.7 ± 0.6. Most of these patients had Child-Pugh C cirrhosis with a disease duration of 8.6 ± 6.6 years. Almost all patients were treated with spironolactone, whereas six patients were on beta-blockers (propranolol or carvedilol). None of the healthy controls had diabetes, arterial hypertension, CAD, peripheral artery disease, chronic kidney disease (III° or IV°), obesity (body mass index > 25 kg/m^2^) or described nicotine abuse. In contrast, in the liver cirrhosis group, six patients showed one and two patients showed at least three of these cardiovascular risk factors. Among the patients with liver cirrhosis who underwent cardiac stress testing (6/10) or echocardiography at rest (8/10), all had normal LV function, all stress tests were normal, and no regional wall motion abnormalities were detected.

### 3.2. Clinical Chemistry

Significant differences in the serum concentrations of bilirubin (*p* < 0.001), AST (*p* = 0.015), albumin (*p* < 0.001), CRP (*p* < 0.001), IL-6 (*p* = 0.008) and TNFα (*p* < 0.001), but not ALT (*p* = 0.123), were observed between the groups. The CRP values exceeded the threshold of 5 mg/dL in all patients with cirrhosis and in only one of the controls. However, the circulating levels of PDGF-BB were reduced by 52% in the cirrhotic group (*p* = 0.001). The levels of creatinine (*p* = 0.062), triglycerides (*p* = 0.554), cholesterol (*p* = 0.342), LDL cholesterol (*p* = 0.457), VEGF (*p* = 0.085), angiotensin II (*p* = 0.356) and bFGF (*p* = 0.134) did not differ between the groups (Table 2).

### 3.3. Effects of Sera on Human Coronary Artery Smooth Muscle Cell Proliferation

**Figure 2:**
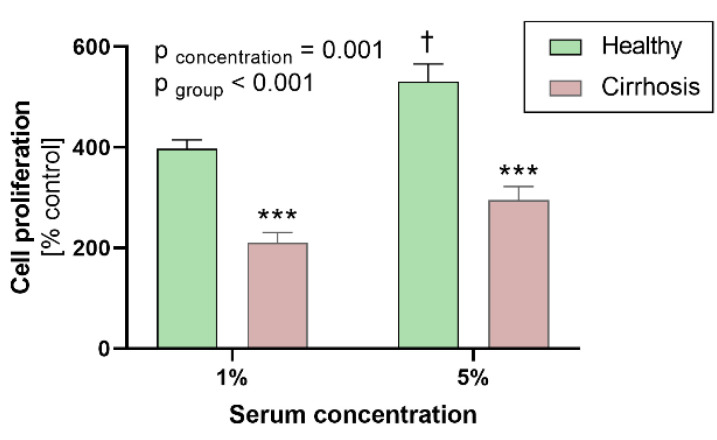
Effects of patient sera on human coronary artery smooth muscle cell proliferation. Effects of 1% and 5% sera from healthy controls and patients with alcoholic liver cirrhosis (each *n* = 10) on proliferation of human coronary artery smooth muscle cells compared to control patients (0.1% serum). (*p*-values from analysis of variance (*p*_concentration_ and *p*_group_) and post hoc analysis: *** *p* ≤ 0.001 vs. healthy; † *p* ≤ 0.05 vs. 1% serum). Data are means ± standard error of the mean.

Exposure to cirrhotic patient serum resulted in a reduction of cell proliferation (210 ± 20% vs. 397 ± 17% (1%) and 295 ± 27% vs. 530 ± 36% (5%), both *p* < 0.001 vs. healthy controls; Figure 2). Exposure to 5% serum increased proliferation compared to 1% serum (*p* = 0.001). This effect remained significant after post-testing in the healthy control group (*p* = 0.019) but not in cirrhotic patients (*p* = 0.254). 

Cell shape and immunofluorescent coloration appeared typical for HCASMC and were consistent over the time period of the experiments. Spreading behavior also did not noticeably change. According to the description of cell phenotypes from Hao et al. [36], cells had spindle-shaped phenotypes.

### 3.4. Effects of Sera on HCASMC Apoptosis

**Figure 3:**
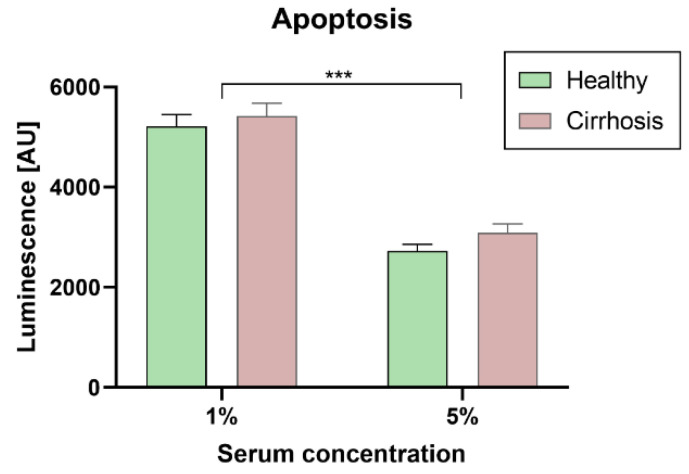
Effects of patient sera on apoptosis of human coronary artery smooth muscle cells. After 48 h of starvation cells were exposed for 48 h under basal (1%) and stimulated (5%) conditions; the rate of apoptosis of human coronary artery smooth muscle did not differ between groups (*n* = 10 per group, *p*-values from two-way analysis of variance: *** *p* ≤ 0.001 1% vs. 5% serum). Data are means ± standard error of the mean.

Stimulation with 5% serum inhibited apoptosis compared to stimulation with 1% serum (*p* < 0.001) (Figure 3). However, for apoptosis no overall group differences between healthy and cirrhotic patients (*p* = 0.160) and no significant interaction between group and serum concentration were observed (*p* = 0.712). 

### 3.5. Effects of Patient Sera on Cell Migration

**Figure 4:**
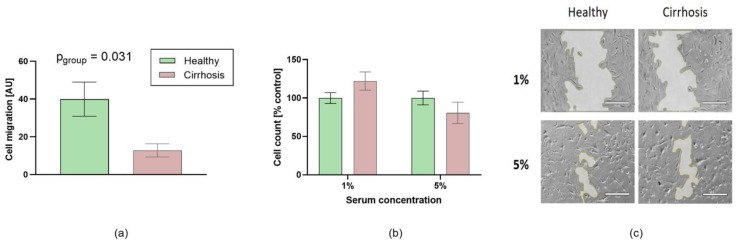
Effects of patient sera on migration of human coronary artery smooth muscle cells. (**a**) Cell migration and (**b**) relative cell count (percent of control) after 48 h are shown. Migration was calculated as explained in Materials and Methods. Cirrhosis markedly reduced cell migration by more than 60% (*n* = 10 per group, *p* < 0.05) (**c**) Representative photographs of cell migration are shown 48 h after scratch induction in 10-fold magnification. The remaining scratch gap is highlighted in the photographs by light grey shading. Scale bars correspond to 0.5 mm length. Data are means ± standard error of the mean.

Cell migration was significantly reduced after exposure to sera from cirrhotic patients (*n* = 10 per groups, *p* < 0.05 vs. healthy controls, Figure 4a,c). Cirrhosis had no effect on cell count (Figure 4b). 

### 3.6. Effects of Sera on HCASMC Gene Expression Analysis

**Figure 5:**
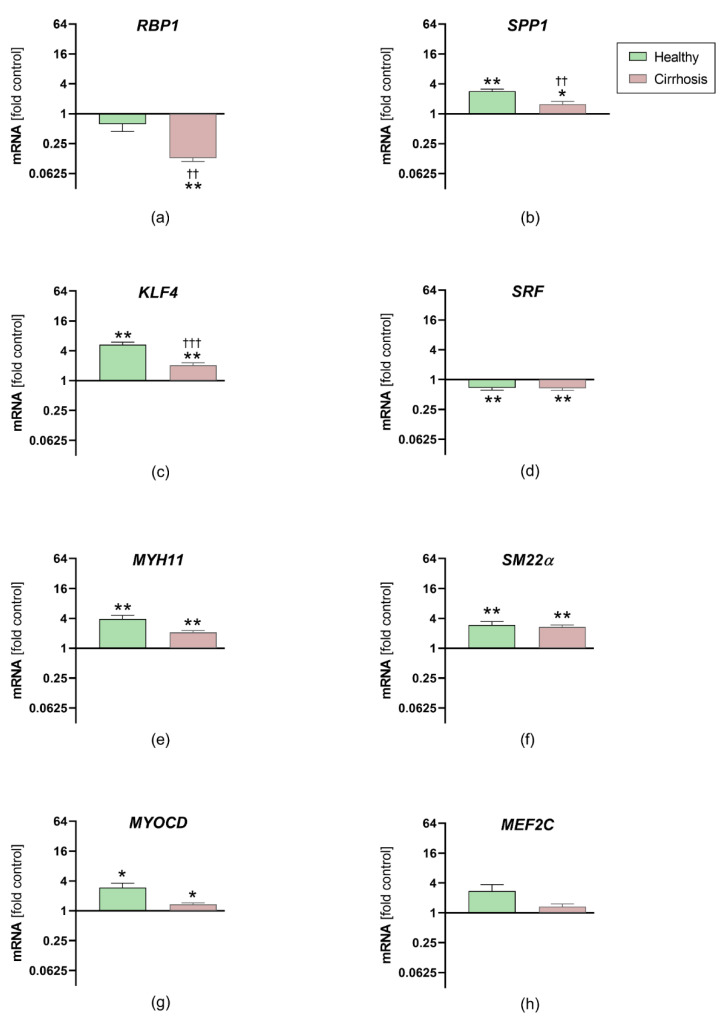
Effects of patient sera on steady-state gene expression levels in human coronary vascular smooth muscle cells. Relative ribonucleic acid expression after 48 h of exposure to 1% serum from either healthy controls or patients with alcoholic liver cirrhosis, compared to that of the negative control (after starving). Retinol binding protein 1 (RBP1), osteopontin (SPP1) and Krüppel-like factor 4 (KLF4) were selected as markers associated with a synthetic phenotype (**a**–**d**). In contrast, myosin heavy chain 11 (MYH11), transgelin (SM22α), myocardin (MYOCD) and myocyte enhancer factor 2C (MEF2C) are known to be associated with a contractile phenotype (**e**–**h**). (one-sample Wilcoxon test; * *p* ≤ 0.05, ** *p* ≤ 0.01 vs. negative control; †† *p* ≤ 0.01, ††† *p* ≤ 0.001 vs. healthy controls). Data are means ± standard error of the means.

After stimulation for 48 h with healthy or cirrhotic sera, coronary vascular smooth muscle cell mRNA expression of SPP1 (*p*_healthy_ = 0.008, *p*_cirrhosis_ = 0.027) and KLF4 (*p*_healthy_ = 0.008, *p*_cirrhosis_ = 0.004) was upregulated in both groups (healthy and cirrhotic), whereas RBP1 was downregulated only in the cirrhotic group (*p*_healthy_ = 0.084, *p*_cirrhosis_ = 0.002). The gene expression levels of RBP1 (*p* = 0.001), SPP1 (*p* = 0.003) and KLF4 (*p* = 0.004) were significantly higher following stimulation with healthy sera than stimulation with cirrhotic sera (Figure 5). No group differences were found for SRF (*p* = 0.853), which was reduced in the cirrhotic (*p* = 0.006) and healthy groups (*p* = 0.004).

The expression levels of MYH11 (*p*_healthy_ = 0.008, *p*_cirrhosis_ = 0.004), SM22α (*p*_healthy_ = 0.004, *p*_cirrhosis_ = 0.002) and MYOCD (*p*_healthy_ = 0.02, *p*_cirrhosis_ = 0.012) were upregulated in both groups after stimulation, with no significant differences between the groups (*p_MYH11_* = 0.114, *p_SM22α_* > 0.999, *p_MYOCD_* = 0.077). Stimulation had no significant effect on MEF2C expression.

## 4. Discussion

This study is the first to demonstrate inhibitory effects of serum from patients with alcoholic liver cirrhosis on proliferation and migration in cultured HCASMC. These effects on cell growth were associated with changes in steady-state gene expression levels indicative of a synthetic smooth muscle cell (SMC) phenotype. 

### 4.1. Effects on Cell Proliferation, Migration and Gene Expression in HCASMC

This study reports inhibition of two key mechanisms relevant to human coronary artery physiology and disease, namely cell proliferation and migration, following exposure to sera obtained from patients with severe alcoholic liver cirrhosis. One of the strengths of this study is that effects were studied in a highly controlled in vitro system using human coronary artery smooth muscle cells. This system is independent of changes in circulating lipids or glucose and independent of blood pressure. In addition, experiments were performed in the absence of any medications. The etiology of hepatic disease in all patients in this study was alcoholic cirrhosis, which is a unique patient sample and has not been studied in the past. Whether similar effects can be obtained with sera from patients with advanced hepatic disease diagnosed with other forms of cirrhosis or other forms of degenerative liver disease, or whether and how responses might be different should be addressed in additional studies. 

The observed effects of patient serum fit well with the observed lower expression of genes indicating a synthetic SMC phenotype (*KLF4*, *SPP1* and *RBP-1*). *KLF4* is a transcription factor involved in phenotypic switching of SMC in response to PDGF, oxidized phospholipids and IL-1ß [37]. *KLF4* resulted in downregulation of *MYOCD* and inhibition of SRF binding to chromatin [38]. Thus, high expression rates are expected to be associated with decreased expression of contractile markers and increased proliferation rates. Only the latter could be observed after stimulation with serum from healthy controls. The downregulation of *SRF* in the present study might reflect a response of this pathway. Alcoholic cirrhosis possibly may increase expression of *KLF4*. In contrast to previous studies, the expression of *MYOCD* and thus *MYH11* and *SM22α* remained high in all groups. *MEF2c* is another regulator of *MYOCD* [39]. The increased levels of *MEF2c* in our study might help to explain the high levels of *MYOCD*, which should be downregulated by *KLF4*. As expected, decreased *SPP1* expression rates were found in slowly proliferating SMC after stimulation with cirrhotic sera. The expression of *RBP1* could be variable in different clones and transient after stimulation [40]. The observed significant downregulation after stimulation with cirrhotic sera is consistent with a contractile phenotype. However, the observed expression of marker genes after stimulation with cirrhotic sera indicate an increased proportion of contractile SMC in culture.

### 4.2. Potential Factors Contributing to Differences in Cell Proliferation and Migration

Proliferation and migration of SMC are influenced by various local and systemic, circulating factors. The balance between growth-stimulating and growth-inhibiting effects and their changes over time determine the total effect. Liver cirrhosis influences a variety of cytokines and vasoactive peptides that affect the function and structure of SMC [41]. 

#### 4.2.1. Circulating Levels of PDGF

The cytokine PDGF is an important factor regulating migration and proliferation of SMCs. Proliferation depends on PDGF levels, whereas migration reaches a maximum at low PDGF concentrations and decreases with higher levels [42]. The observed lower concentration of PDGF in the cirrhotic group could thus be one of the possible factors contributing to the lower proliferation rate, but does not explain the lower migration rate in the cirrhotic group.

#### 4.2.2. Inflammatory Factors

The relevance of systemic inflammation for increased proliferation and migration rates of SMC has been highlighted by Ravindran et al. in the context of ischemia-dependent angiogenesis [43]. In the present study, high circulating levels of CRP, IL-6 and TNF-α in the cirrhotic group are consistent with a chronic inflammatory state, which is typical for end-stage alcoholic liver cirrhosis [44]. However, despite the increased levels of inflammatory factors, sera from patients with alcoholic cirrhosis in this study induced less proliferation and migration than those from healthy controls. Thus, inflammation appears to have only negligible effects on proliferation and migration and is unlikely to be the cause of the observed differences between groups. 

#### 4.2.3. Bilirubin

Bilirubin stimulates apoptosis in SMC [45] and inhibits proliferation both in vitro and in vivo after balloon injury of the carotid artery in a rat model [46]. The antiproliferative effect of bilirubin on SMC was dose-dependent in an in vitro model [46]: A concentration of 2.9 mg/dL, comparable to the serum concentration of our cirrhotic patients, led to a 47% reduction in proliferation. The maximum effect (72% reduction) was observed after the addition of 11.7 mg/dL bilirubin. Nevertheless, relevant effects of bilirubin in the present model are unlikely, as the final concentration of bilirubin in the medium of the cirrhotic group were 0.039 mg/dL (1% serum) and 0.194 mg/dL (5% serum).

### 4.3. Clinical Impact of Alcolholic Cirrhosis on CAD

The results reported in the literature concerning the prevalence of CAD in alcohol-induced liver disease are heterogeneous. Some studies reported a reduction in coronary atherosclerosis [19], whereas others reported an increase and proposed to use alcohol-induced liver disease as an independent risk factor [20]. The MELD score was not reported in the first study and was 14.7 ± 6.7 in the second study and thus slightly lower than the MELD scores in our study. A retrospective multicenter analysis with 17,482 cases described a comparable prevalence of CAD for alcoholic (2.9%) and viral cirrhosis (2.3–2.7%) [18]. These patients had higher MELD scores than those found in other studies (20.5–23.7) [18]. Although the MELD score is being used as an independent predictor of cardiovascular mortality after liver transplantation, it is not correlated with CAD severity [47].

### 4.4. Possible Limitations of the Study

For the present study an in vitro model of atherogenesis studying human coronary artery smooth muscle cell growth was used. As in any in vitro experiment, factors present in vivo such as blood pressure or shear stress, which also contribute to cell migration and proliferation, were absent. Consequently, the in vitro findings may not fully represent the in vivo situation in patients. Subsequent experiments, especially in vivo, are necessary to see whether the results of this study can be confirmed, and to assess the potential role of non-humoral factors and to evaluate whether the findings can be applied to other forms of cirrhosis. Moreover, given the design of the study, it is possible that comorbidities of patients with liver cirrhosis, which are common in this patient population, may have affected the results. Nevertheless, factors in serum from patients with CAD risk factors or overt CAD would be expected to stimulate rather than inhibit SMC proliferation.

## 5. Conclusions

This study provides evidence for the presence of circulating factors in patients with alcoholic liver cirrhosis that inhibit proliferation and migration of human coronary artery vascular smooth muscle cells. In addition, a reduction of circulating levels of PDGF-BB, and increased levels of inflammatory markers such as CRP, IL-6 and TNFα, were observed in patients with alcoholic cirrhosis. Finally, a reduction in the expression of genes associated with differentiation consistent with a synthetic phenotype in coronary smooth muscle cell growth was observed. These findings, suggestive of phenotype switching of SMC mediated by yet unknown circulating factors provide opportunities for further research into pathologies such as coronary artery disease and restenosis following PCI in patients with different forms of cirrhosis.

## Figures and Tables

**Table 1:** Patient characteristics, clinical information, and medications of patients whose serum was used for the experiments. Values are separately presented for the two groups: cirrhotic patients and healthy controls.

Characteristics	Patients with Alcoholic Liver Cirrhosis *n* = 10	Healthy Controls *n* = 10	*p*-Value
Age (years)	50.9 ± 1.5	50.4 ± 2.0	0.847
Women (*n*)	5	8	0.178
MELD score (points)	17.7 ± 0.6	n/a	-
Child-Pugh A, B, C (*n*)	0, 3, 7	n/a	-
Initial diagnosis (years ago)	8.6 ± 6.6	n/a	-
Spironolactone (*n*)	9	0	-
Propranolol/carvedilol (*n*)	6	0	-
Diabetes (*n*)	2	0	-
Hypertension (*n*)	1	0	-
Smoking (*n*)	4	0	-
CAD (*n*)	1	0	-
PAD (*n*)	1	0	-
CKD III° or IV° (*n*)	3	0	-
BMI > 25 kg/m^2^ (*n*)	3	0	-

Data are means ± standard error of the mean. Abbreviations: BMI: body mass index; CAD: coronary artery disease; CKD: chronic kidney disease; MELD Score: Model for End-Stage Liver Disease; n/a: not applicable; PAD: peripheral artery disease.

**Table 2:** Clinical chemistry, cytokine and vasoactive peptide measurements.

Serum Concentration	Patient with Liver Cirrhosis	Healthy Controls	*p*-Value
Bilirubin (mg/dL)	3.87 ± 0.59	0.52 ± 0.09	0.002
AST (U/L)	70 ± 15	25 ± 2	0.015
ALT (U/L)	35 ± 5	25 ± 3	0.123
Creatinine (mg/dL)	1.33 ± 0.15	0.97 ± 0.10	0.062
Albumin (g/dL)	3.0 ± 0.2	5.5 ± 0.2	<0.001
CRP (mg/L)	27.1 ± 4.7	1.5 ± 0.5	<0.001
Cholesterol (mg/dL)	178 ± 23	203 ± 9	0.342
Triglycerides (mg/dL)	126 ± 22	111 ± 13	0.554
LDL cholesterol (mg/dL)	107 ± 11	116 ± 5	0.457
IL-6 (mg/dL)	41.5 ± 11.7	2.1 ± 0.4	0.008
TNFα (pg/mL)	13.3 ± 1.3	4.5 ± 0.3	<0.001
PDGF-BB (pg/mL)	1160 ± 187	2411 ± 265	0.001
VEGF (pg/mL)	52.9 ± 10.2	87.1 ± 15.7	0.085
Angiotensin II (pg/mL)	286.1 ± 60.9	406.0 ± 111.1	0.356
bFGF (pg/mL)	20.09 ± 7.64	5.02 ± 1.42	0.134

Data are means ± standard error of the mean. Abbreviations: AST: Aspartate Aminotransferase; ALT: Alanine Aminotransferase; bFGF: Basic Fibroblast Growth Factor; IL-6: Interleukin 6; LDL: Low-Density Lipoprotein, PDGF-BB: Platelet Derived Growth Factor BB; TNFα: Tumor Necrosis Factor α; VEGF: Vascular Endothelial Growth Factor.

## Data Availability

The data presented in this study are available on request from the corresponding author. The data are not publicly available as they contain information that could compromise the privacy and clinical details of study participants.
